# Platform-specific learning curve and early tibial radiographic agreement of ROSA^®^-assisted medial unicompartmental knee arthroplasty in an experienced high-volume surgeon: a consecutive case series

**DOI:** 10.1007/s00402-026-06497-9

**Published:** 2026-09-02

**Authors:** Stefano Petrillo, Damiano Ardiri, Matteo Marullo, Sergio Romagnoli

**Affiliations:** Prosthetic Surgery Centre, IRCCS Galeazzi Sant’Ambrogio Hospital, Milan, Italy

**Keywords:** Unicompartmental knee arthroplasty, Robotic-assisted surgery, ROSA Partial Knee System, Learning curve, LC-CUSUM, Surgical efficiency, Operative time, Tibial component alignment, Radiographic accuracy

## Abstract

**Purpose:**

To evaluate the platform-specific learning curve and early tibial radiographic agreement of imageless ROSA^®^-assisted medial unicompartmental knee arthroplasty (UKA), with short-term clinical findings assessed exploratorily.

**Methods:**

This retrospective consecutive case series included 30 patients who underwent ROSA^®^-assisted medial UKA with the Persona^®^ Partial Knee implant between January and April 2026 at a high-volume arthroplasty referral centre. A continuous learning-curve cumulative summation (LC-CUSUM) analysis of total surgical duration was performed against an independently defined surgeon-specific historical benchmark derived from institutional operative-register skin-to-skin times for manual UKA performed by the same surgeon (20 ± 4 min). Adequate performance was defined as a mean duration of 25 min, inadequate performance as 30 min, and the reference standard deviation as 4 min. Type I and type II error targets were 0.05 and 0.10, respectively; the decision boundary was calibrated by Monte Carlo simulation over a 30-case monitoring horizon. Robotic time and a supportive comparison of cases 1–5 versus 6–30 were secondary efficiency analyses. Postoperative radiographic measurements were performed by an observer blinded to the planned and robot-validated ROSA^®^ values. Agreement was quantified using Bland–Altman bias and 95% limits of agreement.

**Results:**

Mean robotic time was 14.5 ± 2.4 min, mean non-robotic time was 8.9 ± 3.1 min (range, 4.1–15.6), and mean total surgical duration was 23.4 ± 2.3 min. The continuous LC-CUSUM score crossed the calibrated decision boundary (h = 4.5895) at case 6. Robotic time was 16.0 ± 3.8 min in cases 1–5 and 14.2 ± 2.0 min in cases 6–30 (*p* = 0.787); corresponding total durations were 24.8 ± 3.8 and 23.1 ± 1.9 min (*p* = 0.385). Bland–Altman bias for postoperative minus planned measurements was + 0.13° for tibial coronal alignment and + 0.10° for tibial slope; bias for postoperative minus robot-validated HKA was + 0.34°. All 30 postoperative radiographic measurements were within ± 2° of the planned target for both tibial coronal alignment and slope (100%; exact 95% CI, 88.43%–100%).

**Conclusions:**

The predefined platform-specific temporal-performance standard was reached after six cases by an experienced high-volume UKA surgeon. Tibial planned-to-postoperative deviations were small in this consecutive series, while the findings remain conditional on the specified benchmark and require external validation.

## Introduction

Unicompartmental knee arthroplasty (UKA) is an established treatment option for selected patients with isolated medial compartment osteoarthritis, offering several potential advantages over total knee arthroplasty, including reduced surgical invasiveness, preservation of bone stock and native ligaments, lower blood loss, faster recovery, and more physiological knee kinematics and proprioception [1–8]. When appropriately indicated, medial UKA has shown satisfactory functional outcomes and survivorship; however, its success remains highly dependent on accurate patient selection, implant positioning, and restoration of limb and component alignment [9, 10].

In particular, component malposition and suboptimal alignment have been associated with accelerated polyethylene wear, pain, instability, and early failure after medial UKA [9, 11]. For this reason, considerable effort has been directed toward improving the precision and reproducibility of implant positioning. Robotic assistance has emerged as a valuable adjunct in this setting, as previous comparative studies have shown that robotic-assisted UKA may improve tibial and femoral component positioning compared with conventional techniques [12–15]. Early ROSA-assisted Persona Partial Knee studies have also begun to characterize tibial component placement and short-term outcomes [16, 17], while recent syntheses support broader clinical advantages of robotic-assisted medial UKA compared with conventional implantation [14, 18]. Furthermore, robotic assistance may help less-experienced surgeons achieve a level of accuracy closer to that of high-volume UKA surgeons, thereby potentially reducing technical variability during the adoption phase [19].

Despite these potential advantages, the introduction of any new technology into surgical practice is associated with a learning curve. Studies directly relevant to robotic UKA, Persona Partial Knee, and LC-CUSUM have shown that operative efficiency and reproducibility may change with increasing procedural experience [20–22]. CUSUM and LC-CUSUM provide sequential methods for monitoring performance against an explicit reference standard [23–25]. Within UKA, these analyses indicate that early procedures may differ from later cases in efficiency and consistency [20–22].

Platform-specific evidence must nevertheless be distinguished by study question. Ishimaru et al. described tibial component placement and short-term outcomes with ROSA^®^-assisted Persona Partial Knee arthroplasty [16]. A previous 23-case observational study from our institution primarily evaluated early tibial radiographic accuracy and short-term clinical findings with the same platform and implant [17]. D’Ambrosi et al. evaluated the learning curve of Persona Partial Knee arthroplasty without examining this ROSA^®^ Partial Knee adoption sequence [21]. The present study addresses a different primary question: it analyses the complete initial 30-case chronological sequence using an externally referenced continuous LC-CUSUM, reports robotic, non-robotic, and skin-to-skin operative times, and quantifies agreement among planned, robot-validated, and postoperative measurements using Bland–Altman analysis. Thus, its incremental contribution is the platform-specific adoption and temporal-performance analysis rather than another isolated report of early radiographic accuracy.

Accordingly, the aim of the present study was to characterize the platform-specific learning curve of ROSA^®^-assisted medial UKA in an experienced high-volume surgeon and to quantify planned-to-postoperative tibial radiographic deviations during initial adoption. The radiographic component extends, rather than duplicates, the previous institutional report by evaluating the complete 30-case learning sequence, incorporating planned, robot-validated, and postoperative tibial comparisons, and reporting bias and limits of agreement. The study was not designed to evaluate femoral component position, component sizing, or implant overhang.

## Materials and methods

### Study design

The present study was conducted in accordance with the Strengthening the Reporting of Observational Studies in Epidemiology (STROBE) guidelines. The completed STROBE checklist is provided as Supplementary Material S2.

A retrospective consecutive case-series analysis of a prospectively maintained institutional database was performed on patients who underwent ROSA^®^-assisted medial UKA using the Persona^®^ Partial Knee implant between January and April 2026 at the Joint Replacement Department, [Anonymized Institution], [Anonymized Location]. This investigation was designed primarily as an early platform-specific learning-curve and tibial radiographic accuracy study. Perioperative variables and short-term clinical outcomes were secondary exploratory endpoints. Because the study was single-arm and descriptive, chronological case order represented the main exposure variable; no causal comparison with conventional UKA or other robotic systems was intended.

### Eligibility criteria

Eligibility criteria were defined before data extraction. Inclusion criteria were adults undergoing primary robotic-assisted medial UKA for symptomatic isolated medial compartment knee osteoarthritis, failure of conservative treatment, and availability of complete preoperative, intraoperative, and postoperative radiographic data in the institutional database. Exclusion criteria were revision procedures, active infection, incomplete robotic surgical reports, and missing postoperative radiographs. To minimize selection bias, all consecutive eligible cases performed during the study period were included. Because this was a consecutive early-adoption case series, no patient was excluded from the primary learning-curve or radiographic analyses. A study flow diagram describing patient selection was prepared according to STROBE recommendations and is provided as Supplementary Material S1. No a priori sample size calculation was performed because this was an exploratory early technology evaluation; instead, the study size was determined by the complete consecutive series available during the initial adoption period of the platform at the institution.

### Surgical procedure

All procedures were performed by the senior surgeon using a standardized workflow. ROSA^®^-assisted medial UKA was performed with the Persona^®^ Partial Knee implant through a mid-vastus approach. Following exposure, femoral and tibial tracker pins were positioned and anatomical registration was completed. The imageless platform was used for intraoperative planning, robotic guidance, and validation of tibial coronal alignment and posterior tibial slope. Femoral finishing and final femoral component positioning were completed with the conventional Persona^®^ Partial Knee instrumentation and were not navigated or quantitatively validated as study outputs by the ROSA^®^ Partial Knee workflow. Trial components and definitive implants were inserted after confirmation of alignment, stability, and range of motion. Postoperative rehabilitation was standardized.

### Outcomes

The primary outcome was the platform-specific operative learning curve associated with introduction of the ROSA^®^ Partial Knee System. The principal learning-curve endpoint was total surgical duration according to chronological case order. Total surgical duration was the official skin-to-skin operative time recorded prospectively in the operating-room register by nursing personnel using the operating-room clock, from skin incision to completion of skin closure, in minutes; it included the robotic portion of the procedure. Robotic time, defined as tracker placement, registration, intraoperative planning, robotic bone-cut guidance, and robotic validation, was evaluated as a secondary efficiency outcome. Non-robotic time was calculated as total surgical duration minus robotic time. This study assessed familiarization with a newly introduced partial-knee robotic platform by an experienced high-volume UKA surgeon; it did not assess acquisition of fundamental UKA surgical competence. The surgeon currently performs approximately 250 knee surgical procedures annually, including at least 150 robotic procedures, and has performed approximately 500 manual UKAs over the course of his career. He had extensive prior experience with ROSA^®^-assisted TKA but had performed no robotic UKA before the present consecutive series. The institution was anonymized for peer review and is described as a high-volume arthroplasty referral centre.

Secondary outcomes included tibial radiographic accuracy, perioperative and platform-specific technical events, and short-term clinical outcomes. Planned and final validated tibial values were intraoperative ROSA^®^ outputs; postoperative tibial coronal alignment and posterior slope were measured radiographically. HKA was evaluated separately as a limb-alignment measure. Perioperative, robotic, and radiographic data were complete for all 30 cases. Clinical outcomes were collected in person during the preoperative and 3-month outpatient assessments. VAS pain was recorded on the standard 0–10 scale, where 0 indicated no pain and 10 the worst imaginable pain. VAS and KSS Knee and Function scores were administered and recorded at both time points by the same orthopaedic resident using the standard scoring forms. Because recruitment occurred during a four-month surgical window, paired clinical follow-up had matured only for the first 10 chronological cases at data extraction; missing clinical follow-up for the remaining cases was therefore systematic and time-dependent rather than random. Femoral component position, sizing, and overhang were not accuracy endpoints.

### Radiographic assessment

Postoperative radiographic assessment was performed at the first standardized radiographic follow-up, usually approximately 3 months after surgery. Full-length weight-bearing anteroposterior lower-limb radiographs and true lateral knee radiographs were obtained according to the institutional protocol. HKA was measured on full-length radiographs and reported as degrees of varus from neutral mechanical alignment. Postoperative tibial coronal alignment was measured relative to the tibial mechanical axis, with varus reported as positive, and posterior tibial slope was measured relative to the proximal tibial anatomical axis on the true lateral radiograph. Planned and final validated values were taken directly from the ROSA^®^ report using the system’s standard definitions. No separate radiographic definition was assigned to these intraoperative ROSA^®^ outputs.

All postoperative radiographic measurements were performed in Sectra IDS7, version 27.2 (Sectra AB, Linköping, Sweden), by one experienced observer who was blinded to the planned and final robot-validated ROSA^®^ values. Robotic reports and radiographic images were reviewed in separate sessions, and the ROSA^®^ values were not available to the observer during radiographic measurement. ROSA^®^ planned and final validated tibial values were recorded in the 0.5° increments provided by the robotic interface. Postoperative tibial coronal alignment and posterior tibial slope were measured independently in Sectra using the angular measurement tool employed for these component measurements, which displayed whole-degree values (1° resolution). HKA was measured separately on full-length weight-bearing radiographs using a different Sectra measurement tool that allowed decimal-degree readings. Source measurements were retained at their native displayed resolution rather than assigned artificial additional precision; calculated tibial deviations therefore occur in 0.5° increments when a 1° Sectra value is compared with a 0.5° ROSA value. Consequently, repeated 0.0° tibial deviations indicate agreement at the available measurement resolution and should not be interpreted as exact sub-degree physical identity between modalities. Bland–Altman bias, confidence intervals, and limits of agreement were reported to 0.01° because they are cohort-level statistical estimates derived from the complete paired dataset. Intraobserver reliability was evaluated in a subset of 15 cases: the same observer repeated HKA, tibial coronal alignment, and tibial slope measurements after a 21-day washout and remained blinded to the robotic values. Reliability was estimated using a two-way mixed-effects, absolute-agreement, single-measure ICC. Interobserver reliability was not assessed because a second independent radiographic observer was not available.

### Learning-curve assessment

A continuous learning-curve cumulative summation (LC-CUSUM) analysis was performed on total surgical duration in chronological case order [22, 24, 25]. The null hypothesis represented inadequate performance with a mean duration µ1 = 30 min, whereas the alternative hypothesis represented adequate performance with µ0 = 25 min. These thresholds were defined independently of the observed robotic sequence from the surgeon-specific historical manual-UKA benchmark documented in the institutional operating-room register. The benchmark used the same skin-to-skin definition as the present study from skin incision to completion of skin closure and was 20 ± 4 min. An additional 5 min was considered an acceptable robotic overhead, whereas an additional 10 min was defined as inadequate. The reference standard deviation was σ = 4 min, and the reference value was k = (µ0 + µ1)/2 = 27.5 min. For case i with observed duration xi, the log-likelihood increment was wi = [(µ1 − µ0)/σ²][(µ0 + µ1)/2 − xi], and the learning score was Si = max(0, Si − 1 + wi), with S0 = 0. Adequate performance was signalled when the score first reached the decision boundary h. Type I and type II error targets were α = 0.05 and β = 0.10. The boundary was calibrated over the prespecified 30-case monitoring horizon using 2,000,000 Monte Carlo sequences generated under the inadequate-performance distribution, producing h = 4.5895 and an estimated false-positive probability of 0.0500. Under the adequate-performance distribution, the corresponding estimated power was 0.9998. This externally referenced LC-CUSUM replaced the cohort-mean-centred CUSUM, whose crossing point is mathematically dependent on the analysed series.

Robotic time was analysed descriptively as a secondary platform-specific metric. In addition, cases 1–5 were compared with cases 6–30, thereby aligning the supportive grouped analysis with the first LC-CUSUM boundary crossing at case 6. This grouped comparison remained secondary and was not used to define or recalibrate the proficiency threshold.

### Statistical analysis

Tibial radiographic accuracy was analysed using postoperative deviation from the corresponding planned ROSA^®^ tibial target. The prespecified clinically acceptable margin was ± 2°, with ± 3° reported secondarily. Exact Clopper–Pearson confidence intervals were calculated for the proportion within each margin. Bland–Altman analysis [26] was performed for seven prespecified comparisons: validated minus planned, postoperative minus planned, and postoperative minus validated values for tibial coronal alignment and posterior tibial slope, together with postoperative minus validated HKA. For each comparison, mean bias, standard deviation of the paired differences, 95% confidence interval of the bias, and 95% limits of agreement were calculated. Confidence intervals for the limits of agreement used the standard error SDdiff × √[1/*n* + 1.96²/(2(*n* − 1))] with the t distribution. Positive differences indicate that the second measurement was more varus for coronal/HKA values or had a greater posterior slope. HKA agreement was reported as a secondary limb-alignment analysis. No statistical inference was performed for femoral position, sizing, or overhang because these variables were not collected and were outside the validated tibial outputs assessed in this study.

Comparisons between cases 1–5 and cases 6–30 were performed using the exact two-sided Mann–Whitney U test for continuous variables and Fisher’s exact test for categorical variables, as appropriate. The final robot-validated versus postoperative HKA comparison was evaluated with a two-sided paired t-test. Paired clinical outcomes in the chronological 10-case subset were evaluated using exact two-sided Wilcoxon signed-rank tests. Grouped radiographic analyses used the same definitions as the complete-cohort framework and remained secondary to the paired agreement analyses. No formal sensitivity analyses or multivariable adjusted models were performed because the study was designed as a descriptive early-adoption series rather than a causal comparative analysis. Continuous data are reported as mean ± standard deviation, all radiographic comparisons include 30 paired cases, and statistical significance was set at *p* < 0.05. Case-level operative timing and LC-CUSUM values are provided as Supplementary Data S3, and case-level planned, robot-validated, and postoperative tibial measurements are provided as Supplementary Data S4.

## Results

### Patient demographics and perioperative data

Thirty consecutive patients were screened and included. Operative timing, intraoperative robotic records, perioperative variables, technical-event fields, preoperative HKA, and postoperative radiographic measurements were complete for all 30/30 cases. No patient was excluded and no primary outcome value was missing. Three-month clinical data were available for exactly the first 10 consecutive cases. Because the study operations occurred between January and April 2026, the remaining 20 cases had not reached mature 3-month follow-up at the time of extraction. Clinical missingness was therefore systematic and chronological, not random. All clinical outcomes were complete within the 10-case subset; there were no isolated missing clinical data points and no imputation was performed.

The cohort comprised 18 men and 12 women, with a mean age of 67.3 ± 9.1 years and a mean body mass index of 28.8 ± 4.1 kg/m². Preoperative HKA was available in all 30 cases and averaged 5.4 ± 3.6° varus. Mean preoperative hemoglobin was 14.1 ± 1.2 g/dL, and mean hemoglobin drop was 0.9 ± 0.4 g/dL. Mean robotic time was 14.5 ± 2.4 min, mean non-robotic time was 8.87 ± 3.08 min (range, 4.07–15.60), and mean total surgical duration was 23.4 ± 2.3 min (Table 1). Across all 30 procedures, there were no registration failures, tracker-pin complications, intraoperative plan revisions, robotic aborts, conversions to conventional instrumentation, or other recorded complications. Cohort and perioperative characteristics are summarized in Table 1.

**Table 1:** Demographic and perioperative characteristics

Variable	Value
Patients, n	30
Age, years	67.3 ± 9.1
Sex, male/female	18/12
BMI, kg/m²	28.8 ± 4.1
Side, right/left	15/15
Robotic time, min	14.4 (13.3–15.4)
Total surgical duration, min	23.4 ± 2.3
Non-robotic time, min	8.9 ± 3.1; range 4.1–15.6
Preoperative HKA, ° varus	5.2 (2.6–7.9)
Preoperative haemoglobin, g/dL	14.1 ± 1.2
Haemoglobin decrease, g/dL	0.9 ± 0.4

Normally distributed continuous variables are mean ± SD; non-normally distributed variables are median (IQR), according to Shapiro–Wilk testing. HKA, hip–knee–ankle angle

### Learning-curve analysis

**Fig. 1:**
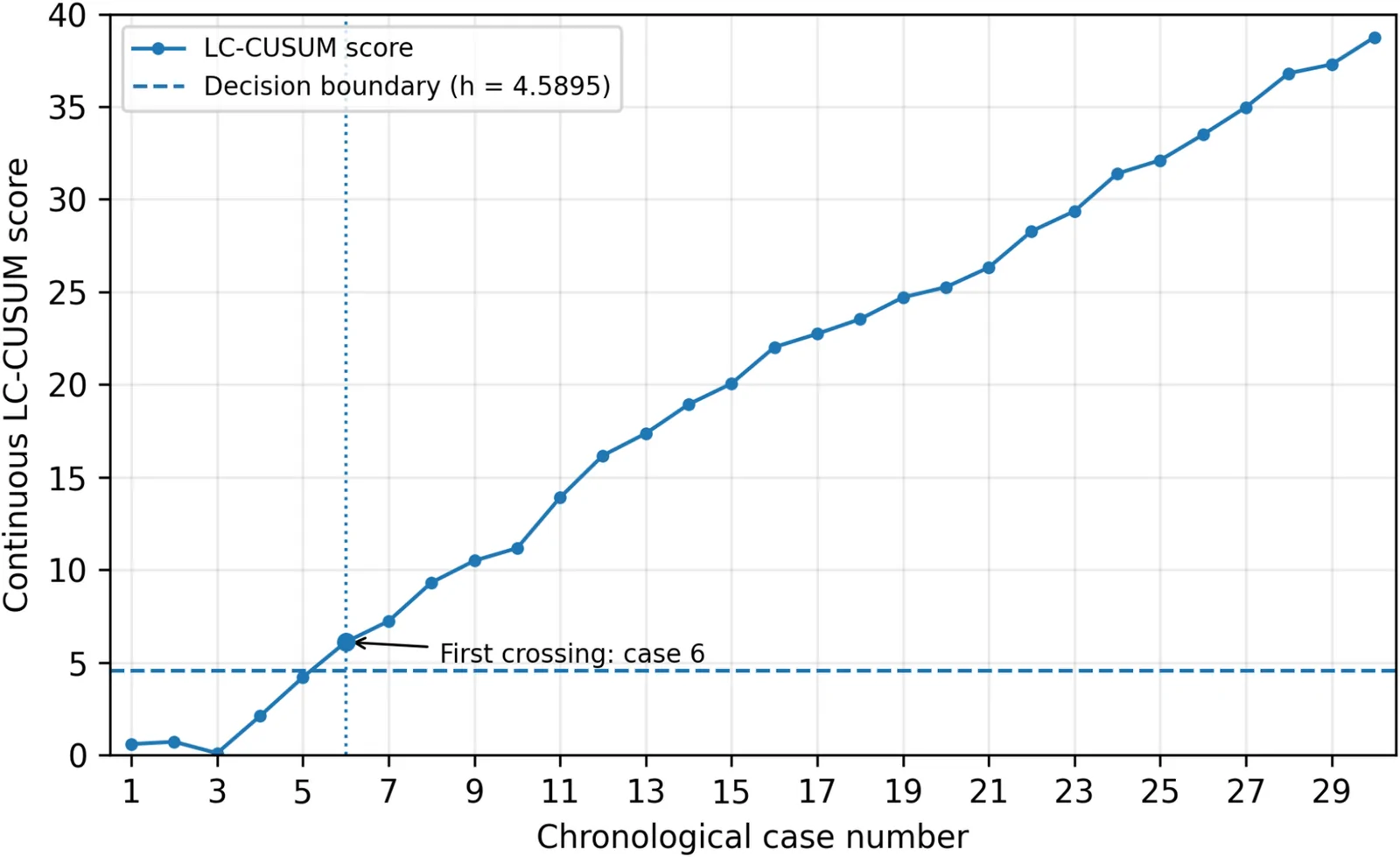
Continuous LC-CUSUM of total surgical duration. The decision boundary (h = 4.5895) was crossed at case 6, indicating attainment of the predefined platform-specific operative-performance standard. µ0 = 25 min; µ1 = 30 min; σ = 4 min; α = 0.05; monitoring horizon = 30 cases

Continuous LC-CUSUM analysis of total surgical duration crossed the calibrated decision boundary of h = 4.5895 at case 6 (Fig. 1). The score was 4.1979 after case 5 and increased to 6.0990 after case 6, thereby rejecting the null hypothesis of inadequate temporal performance. The score remained above the boundary throughout the remaining sequence and reached 38.7448 at case 30. Thus, relative to the independently defined historical benchmark, the predefined platform-specific operative-performance standard was attained after six cases.

In the supportive grouped comparison aligned with the first LC-CUSUM boundary crossing, robotic time was 16.0 ± 3.8 min in cases 1–5 and 14.2 ± 2.0 min in cases 6–30 (*p* = 0.787). Total surgical duration was 24.8 ± 3.8 min and 23.1 ± 1.9 min, respectively (*p* = 0.385). Non-robotic time was 8.84 ± 3.91 min in cases 1–5 and 8.88 ± 2.98 min in cases 6–30 (*p* = 0.914). These secondary comparisons were not used to determine the LC-CUSUM crossing (Table 2).

**Table 2:** LC-CUSUM parameters and supportive grouped comparison

Variable/parameter	Cases 1–5	Cases 6–30	*p*-value/interpretation
Adequate mean (µ0), min	25	–	Prespecified
Inadequate mean (µ1), min	30	–	Prespecified
Reference SD, min	4	–	Prespecified
Decision boundary, h	4.5895	–	Crossed at case 6
Robotic time, min	14.3 (13.9–15.2)	14.4 (13.0–15.4)	0.787
Total surgical duration, min	25.6 (21.1–27.1)	23.7 (21.4–24.1)	0.385
Non-robotic time, min	7.3 (6.9–11.3)	8.7 (6.9–10.7)	0.914
Absolute coronal deviation from plan, °	0.0 (0.0–0.0)	0.0 (0.0–0.0)	1.000
Absolute slope deviation from plan, °	0.0 (0.0–0.5)	0.0 (0.0–0.5)	0.589

Grouped continuous results are median (IQR) and were compared using the exact two-sided Mann–Whitney U test. This comparison is secondary to the continuous LC-CUSUM

### Tibial radiographic accuracy and HKA agreement

Mean planned ROSA^®^, final robot-validated, and postoperative radiographic tibial coronal values were 1.93 ± 0.37°, 2.03 ± 0.49°, and 2.07 ± 0.58° of varus, respectively. The planned-to-validated difference (+ 0.10 ± 0.58°; mean absolute difference, 0.47 ± 0.35°) describes intraoperative execution of the plan and is reported separately from radiographic accuracy.

Mean planned ROSA^®^, final robot-validated, and postoperative radiographic posterior tibial slope values were 4.77 ± 0.45°, 4.90 ± 0.67°, and 4.87 ± 0.73°, respectively. The mean absolute postoperative deviation from the planned target was 0.37 ± 0.47°. The planned-to-validated difference (+ 0.13 ± 0.78°; mean absolute difference, 0.63 ± 0.45°) describes intraoperative execution rather than a radiographic outlier endpoint. The complete planned, robot-validated, and postoperative measurements are reported in Table 3.

**Table 3:** Complete planned, robot-validated, and postoperative measurements

Parameter	Preoperative/planned mean ± SD	Preoperative/planned median (IQR)	Robot-validated mean ± SD	Robot-validated median (IQR)	Postoperative mean ± SD	Postoperative median (IQR)
HKA, ° varus	5.4 ± 3.6	5.2 (2.6–7.9)	2.2 ± 0.7	2.0 (1.7–2.6)	2.5 ± 0.9	2.5 (2.1–3.2)
Tibial coronal alignment, ° varus	1.9 ± 0.4	2.0 (2.0–2.0)	2.0 ± 0.5	2.0 (1.5–2.5)	2.1 ± 0.6	2.0 (2.0–2.0)
Posterior tibial slope, °	4.8 ± 0.5	5.0 (4.5–5.0)	4.9 ± 0.7	5.0 (4.5–5.5)	4.9 ± 0.7	5.0 (5.0–5.0)

HKA has no planned ROSA^®^ field; the first HKA columns therefore show preoperative radiographic HKA. Both mean ± SD and median (IQR) are supplied to preserve Bland–Altman-compatible means while transparently describing non-normal distributions. Postoperative tibial coronal and slope source measurements were recorded at 1° resolution in Sectra, whereas HKA was obtained with a separate Sectra measurement tool allowing decimal-degree readings

**Fig. 2:**
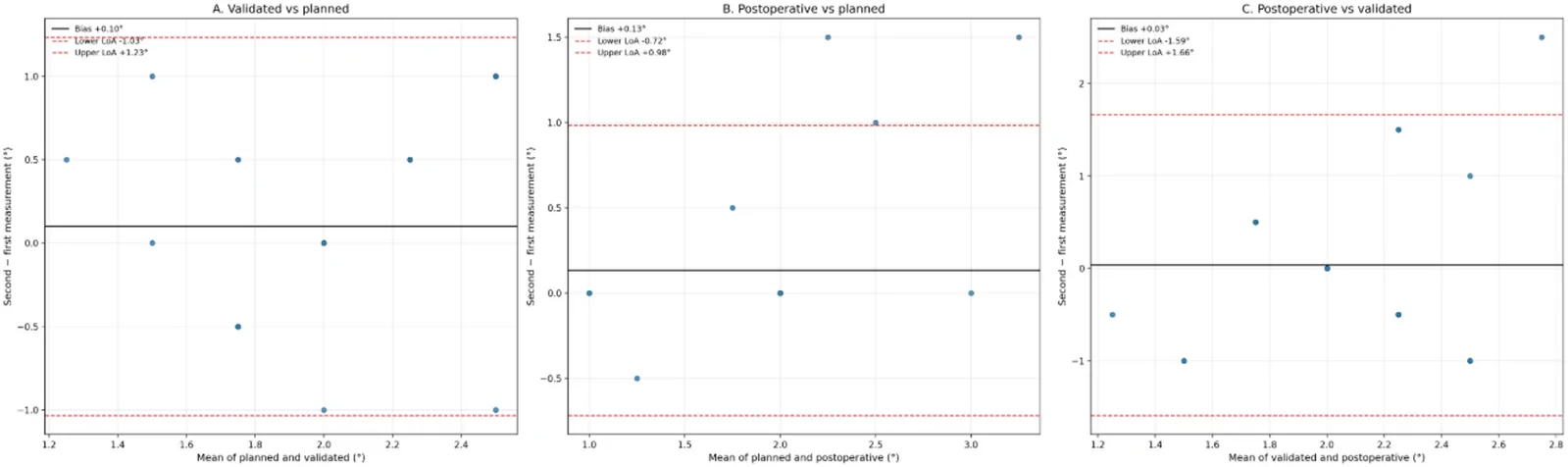
Bland–Altman plots for tibial coronal alignment: (**A**) Final robot-validated minus planned, (**B**) Postoperative minus planned, and (**C**) Postoperative minus final robot-validated measurements. Solid lines show mean bias; Dashed lines show 95% limits of agreement

**Fig. 3:**
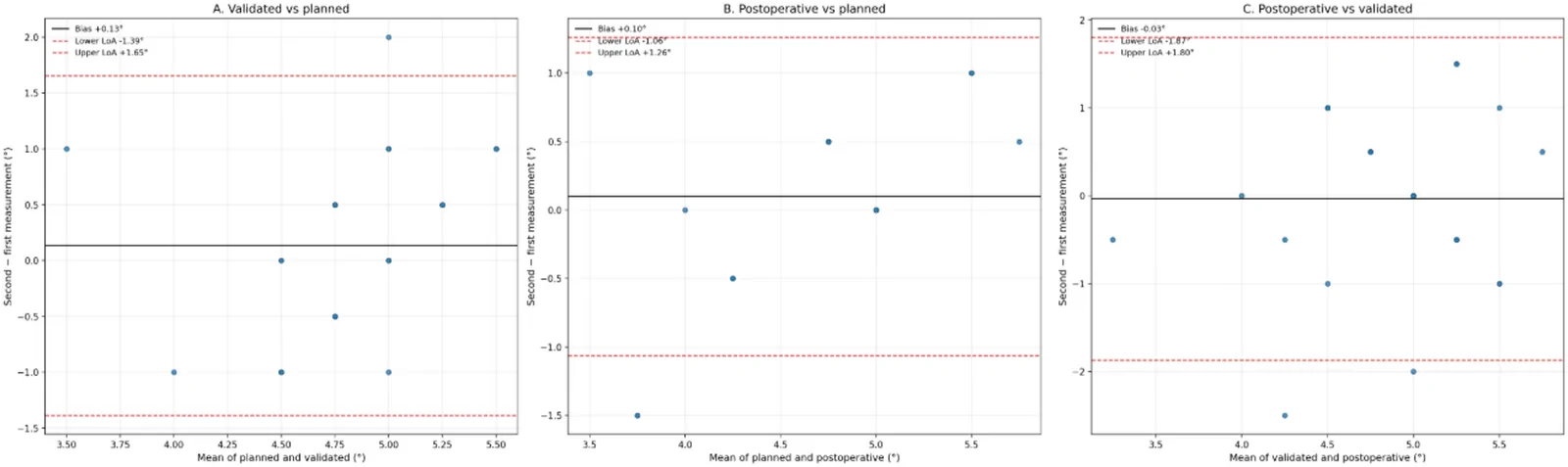
Bland–Altman plots for posterior tibial slope: (**A**) final robot-validated minus planned, (**B**) postoperative minus planned, and (**C**) postoperative minus final robot-validated measurements. Solid lines show mean bias; Dashed lines show 95% limits of agreement

**Fig. 4:**
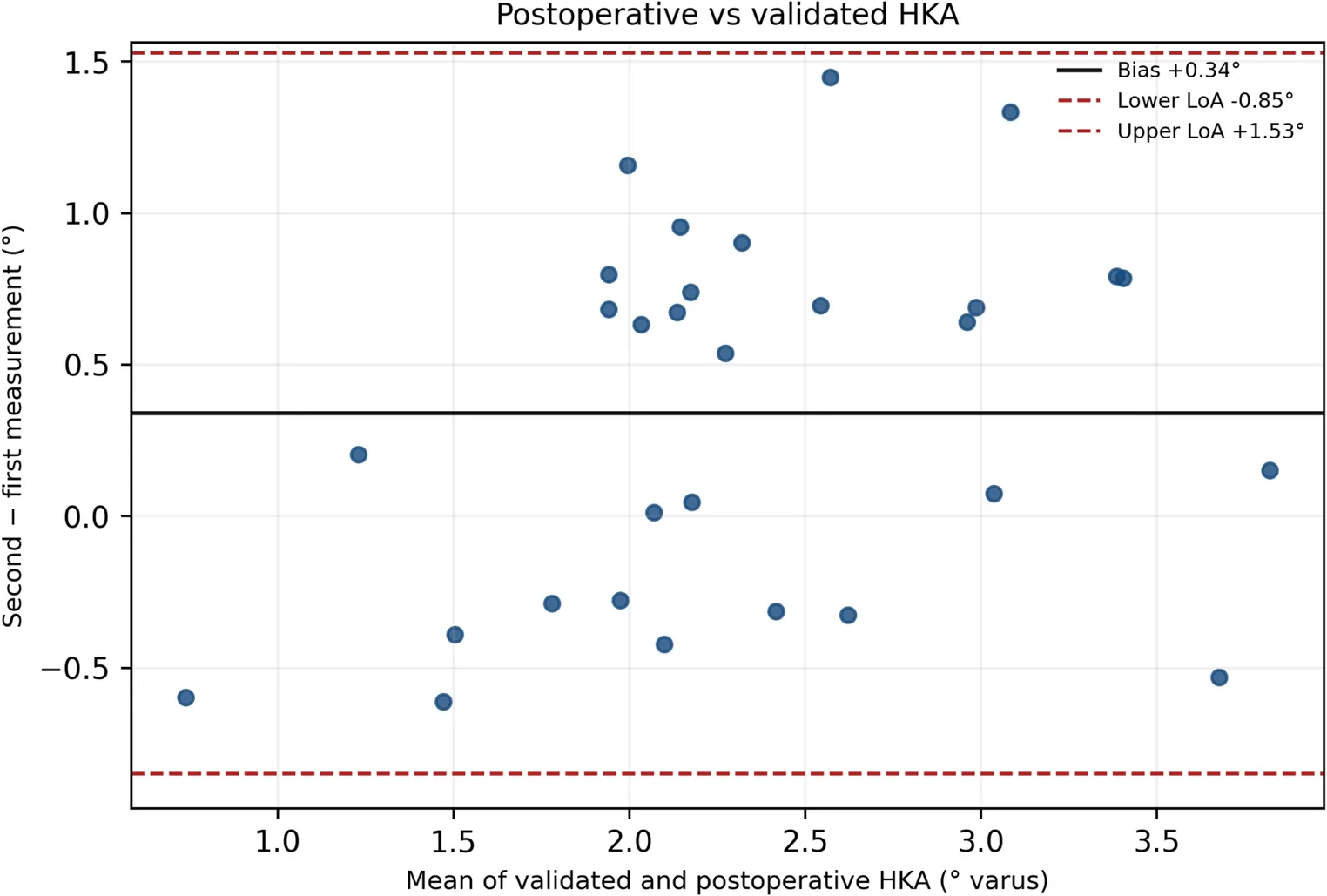
Bland–Altman plot for postoperative minus final robot-validated HKA. The solid line shows mean bias; dashed lines show 95% limits of agreement

Bland–Altman results are reported throughout as the second measurement minus the first. For tibial coronal alignment, validated minus planned values showed a bias of + 0.10° (SD of differences, 0.58°; 95% CI of bias, − 0.12° to + 0.32°) with 95% limits of agreement (LoA) of − 1.03° to + 1.23°. Postoperative minus planned values showed a bias of + 0.13° (SD, 0.43°; 95% CI, − 0.03° to + 0.30°; LoA, − 0.72° to + 0.98°), while postoperative minus validated values showed a bias of + 0.03° (SD, 0.83°; 95% CI, − 0.28° to + 0.34°; LoA, − 1.59° to + 1.66°). For posterior tibial slope, validated minus planned values showed a bias of + 0.13° (SD, 0.78°; 95% CI, − 0.16° to + 0.42°; LoA, − 1.39° to + 1.65°). Postoperative minus planned values showed a bias of + 0.10° (SD, 0.59°; 95% CI, − 0.12° to + 0.32°; LoA, − 1.06° to + 1.26°), and postoperative minus validated values showed a bias of − 0.03° (SD, 0.94°; 95% CI, − 0.38° to + 0.32°; LoA, − 1.87° to + 1.80°). Final robot-validated and postoperative HKA averaged 2.18 ± 0.69° and 2.52 ± 0.85° varus, respectively. Postoperative minus validated HKA showed a bias of + 0.34° (SD, 0.61°; 95% CI, + 0.11° to + 0.57°), with LoA of − 0.85° to + 1.53°. The paired HKA difference was statistically significant (paired t-test, *p* = 0.005). Confidence intervals for each lower and upper LoA are provided in Table 4. In the 15-case blinded repeat-measurement subset, intraobserver reliability was excellent for HKA (ICC = 0.92) and good for tibial coronal alignment (ICC = 0.89) and tibial slope (ICC = 0.86). The corresponding agreement plots are shown in Figs. 2, 3 and 4.

**Table 4:** Complete Bland–Altman agreement analysis

Comparison (second−first)	Bias, °	SDdiff, °	95% CI of bias, °	Lower LoA, ° (95% CI)	Upper LoA, ° (95% CI)	*n*
Coronal: validated−planned	+ 0.10	0.58	−0.12 to + 0.32	−1.03 (− 1.41 to − 0.66)	+ 1.23 (+ 0.86 to + 1.61)	30
Coronal: postoperative−planned	+ 0.13	0.43	−0.03 to + 0.30	−0.72 (− 1.00 to − 0.44)	+ 0.98 (+ 0.70 to + 1.26)	30
Coronal: postoperative−validated	+ 0.03	0.83	−0.28 to + 0.34	−1.59 (− 2.13 to − 1.06)	+ 1.66 (+ 1.12 to + 2.20)	30
Slope: validated−planned	+ 0.13	0.78	−0.16 to + 0.42	−1.39 (− 1.89 to − 0.89)	+ 1.65 (+ 1.15 to + 2.16)	30
Slope: postoperative−planned	+ 0.10	0.59	−0.12 to + 0.32	−1.06 (− 1.45 to − 0.68)	+ 1.26 (+ 0.88 to + 1.65)	30
Slope: postoperative−validated	−0.03	0.94	−0.38 to + 0.32	−1.87 (− 2.47 to − 1.27)	+ 1.80 (+ 1.20 to + 2.41)	30
HKA: postoperative−validated	+ 0.34	0.61	+ 0.11 to + 0.57	−0.85 (−1.24 to −0.46)	+ 1.53 (+ 1.14 to + 1.92)	30

Differences are calculated as the second measurement minus the first. Positive values indicate greater varus for coronal/HKA measurements or greater posterior slope. *SDdiff* standard deviation of paired differences, *LoA* limits of agreement. ROSA^®^ tibial source values were recorded in 0.5° increments, postoperative tibial Sectra values at 1° resolution, and HKA using a separate decimal-degree Sectra measurement tool. Bland–Altman agreement estimates are reported to 0.01° as cohort-level statistical estimates

### Absolute deviation from target and outliers

For the primary radiographic accuracy endpoints, all 30 postoperative coronal measurements and all 30 postoperative slope measurements were within ± 2° of the planned ROSA^®^ target: 100% for each endpoint (exact Clopper–Pearson 95% CI, 88.43%–100%). Equivalently, the observed > 2° radiographic-outlier rate was 0/30, or 0% (exact 95% CI, 0%–11.57%). All 30 cases were also within ± 3° for both parameters (100%; exact 95% CI, 88.43%–100%). Secondary postoperative HKA deviation from the final robot-validated value was also within ± 2° in all 30 cases (100%; exact 95% CI, 88.43%–100%). Absolute deviations and exact confidence intervals are summarized in Table 5.

**Table 5:** Absolute deviation and cases within prespecified margins

Comparison	Mean ± SD, °	Median (IQR), °	Range, °	Cases within ± 2°, *n*/*N* (%)	Exact 95% CI	Cases within ± 3°, *n*/*N* (%)	Exact 95% CI
HKA: validated to postoperative	0.6 ± 0.4	0.6 (0.3–0.8)	0.0–1.4	30/30 (100%)	88.43–100.00%	30/30 (100%)	88.43–100.00%
Tibial coronal: planned to postoperative	0.2 ± 0.4	0.0 (0.0–0.0)	0.0–1.5	30/30 (100%)	88.43–100.00%	30/30 (100%)	88.43–100.00%
Tibial slope: planned to postoperative	0.4 ± 0.5	0.0 (0.0–0.5)	0.0–1.5	30/30 (100%)	88.43–100.00%	30/30 (100%)	88.43–100.00%

Confidence intervals are exact two-sided Clopper–Pearson intervals. The primary outlier definition was > 2°; a value equal to 2.0° is within the prespecified ± 2° margin

Descriptively, mean absolute planned-to-postoperative deviation was 0.10 ± 0.22° in cases 1–5 and 0.18 ± 0.45° in cases 6–30 for tibial coronal alignment (*p* = 1.000), and 0.20 ± 0.27° and 0.40 ± 0.50°, respectively, for slope (*p* = 0.589). Secondary validated-to-postoperative HKA deviation was 0.47 ± 0.18° and 0.61 ± 0.38°, respectively (*p* = 0.416). These non-significant secondary comparisons do not demonstrate maintenance of radiographic accuracy or equivalence between phases.

### Early clinical outcomes

Short-term clinical findings at approximately 3 months are reported for the first 10 chronological cases, which were the only cases with mature paired clinical data at extraction because recruitment occurred during a four-month surgical window. Baseline and 3-month VAS, KSS Knee, KSS Function, extension deficit, and maximum flexion were complete for all 10 patients. VAS and KSS were collected in person and scored by the same orthopaedic resident at the preoperative and 3-month visits. Accordingly, each paired analysis used *n* = 10; there were no outcome-specific missing observations and no imputation.

In this chronological subset (*n* = 10), VAS improved from 8.0 ± 0.8 preoperatively to 1.2 ± 1.5 at 3 months (*p* = 0.002). At 3 months, VAS was 3 in four patients and 0 in the remaining six patients. KSS Knee improved from 38.2 ± 2.9 to 88.8 ± 2.1 (*p* = 0.002), while KSS Function improved from 46.0 ± 7.0 to 97.0 ± 4.8 (*p* = 0.002). Extension deficit decreased from 3.5 ± 4.7° to 0.5 ± 1.6° (*p* = 0.037), and maximum flexion increased from 116.0 ± 14.3° to 123.0 ± 9.5° (*p* = 0.131). These analyses are descriptive and exploratory because the subset was determined by chronological follow-up maturity rather than representing the entire cohort. The complete clinical results are reported in Table 6. Outcome-specific data availability is summarized in Table 7.

**Table 6:** Exploratory clinical outcomes in the chronological 10-case subset

Outcome	Preoperative mean ± SD	Preoperative median (IQR)	3-month mean ± SD	3-month median (IQR)	Paired *n*	Exact *p*-value
VAS	8.0 ± 0.8	8.0 (7.4–8.2)	1.2 ± 1.5	0.0 (0.0–3.0)	10	0.002
KSS Knee	38.2 ± 2.9	38.6 (37.3–39.3)	88.8 ± 2.1	89.2 (87.9–89.8)	10	0.002
KSS Function	46.0 ± 7.0	47.0 (41.2–50.7)	97.0 ± 4.8	97.4 (95.6–99.5)	10	0.002
Extension deficit, °	3.5 ± 4.7	2.3 (1.0–3.5)	0.5 ± 1.6	0.0 (0.0–0.0)	10	0.037
Maximum flexion, °	116.0 ± 14.3	112.2 (106.1–121.0)	123.0 ± 9.5	124.9 (117.9–126.7)	10	0.131

VAS, KSS, and range-of-motion outcomes were collected in person by the same orthopaedic resident. P-values are from exact paired Wilcoxon signed-rank analyses. At 3 months, VAS was 0 in six patients and 3 in four patients

**Table 7:** Data availability matrix. Clinical availability was determined by chronological maturation of 3-month follow-up

Outcome/domain	Available, *n*/*N*	Availability pattern
Demographic variables	30/30	Complete
Operative timing: robotic, non-robotic, total	30/30	Complete
Perioperative hemoglobin and hemoglobin drop	30/30	Complete
Preoperative HKA	30/30	Complete
ROSA^®^ planned and validated tibial parameters	30/30	Complete
Postoperative HKA, tibial coronal alignment, and slope	30/30	Complete
Platform-specific technical-event fields	30/30	Complete
VAS: baseline and 3 months	10/30	First 10 chronological cases
KSS Knee: baseline and 3 months	10/30	First 10 chronological cases
KSS Function: baseline and 3 months	10/30	First 10 chronological cases
Extension deficit: baseline and 3 months	10/30	First 10 chronological cases
Maximum flexion: baseline and 3 months	10/30	First 10 chronological cases
Radiographic intraobserver reliability	15/30	Reliability subset; 21-day washout

### Summary of main findings

Continuous LC-CUSUM reached the predefined platform-specific temporal-performance boundary at case 6. Secondary grouped comparisons showed no statistically significant phase differences and were interpreted descriptively. All postoperative tibial coronal and slope measurements were within ± 2° of their planned ROSA^®^ targets in this 30-case series. No platform-specific technical event or other complication was observed. Clinical findings were restricted to the first 10 chronological cases and remain exploratory.

## Discussion

In this series, the prespecified LC-CUSUM temporal-performance boundary was crossed at case 6. This result describes platform-specific familiarization by an experienced high-volume UKA surgeon; it does not indicate acquisition of fundamental UKA competence or define a universal proficiency threshold. Postoperative radiographs showed small deviations from planned tibial coronal alignment and posterior slope across the observed sequence.

The radiographic endpoint was deliberately restricted to the tibial parameters directly planned and validated within the studied ROSA^®^ workflow. Planned and validated tibial values are intraoperative ROSA^®^ outputs, whereas achieved tibial alignment and slope are postoperative radiographic measurements. Robot-validated versus postoperative Bland–Altman results therefore characterize tibial inter-modality agreement. They do not establish accuracy of the femoral component or of the implant construct as a whole.

The findings should be interpreted in relation to existing platform-specific evidence. Ishimaru et al. [16] described tibial component placement and short-term outcomes with ROSA^®^-assisted Persona Partial Knee arthroplasty, while our previous institutional study [17] focused on early tibial radiographic accuracy. The present contribution is the complete initial 30-case adoption sequence, externally benchmarked LC-CUSUM, separation of robotic and non-robotic time, and formal agreement analysis for tibial ROSA^®^ outputs. Because there was no manual control group and femoral position, sizing, and overhang were not assessed, the data cannot establish superiority over conventional UKA or comprehensive component-position accuracy.

All 30 postoperative coronal and slope measurements were within the prespecified ± 2° margin relative to plan. The exact 95% confidence interval for 30/30 observations was 88.43%–100%; equivalently, 0/30 observed outliers remains compatible with a true outlier proportion as high as 11.57%. These estimates describe only the observed tibial measurements in this series. The small sample produces uncertainty around both the proportion within the margin and the Bland–Altman limits, and does not establish perfect accuracy in the underlying population. Bland–Altman limits describe individual ROSA^®^-to-radiograph agreement without implying causal or comparative superiority.

The + 0.34° postoperative-minus-validated HKA bias (95% CI, + 0.11° to + 0.57°) indicates a small systematic shift toward a more varus value on postoperative weight-bearing radiographs rather than an absence of inter-modality difference. The LoA of − 0.85° to + 1.53° remained relatively narrow in this series. This pattern may reflect differences between intraoperative and weight-bearing assessment, limb positioning, radiographic projection, landmark identification, and the coordinate systems used by ROSA^®^ and long-leg radiography. Accordingly, validated ROSA^®^ HKA and postoperative radiographic HKA should be regarded as closely related but not interchangeable measurements.

The LC-CUSUM result is conditional on an adequate mean of 25 min, an inadequate mean of 30 min, a reference standard deviation of 4 min, the stated error targets, and the 30-case monitoring horizon. These inputs were derived independently from the surgeon-specific manual-UKA operative-time benchmark documented in the institutional operating-room register, using the same skin-to-skin definition as the present study (20 ± 4 min), with allowance for robotic overhead. The surgeon’s career experience of approximately 500 manual UKAs describes his procedural background and is distinct from the operative-register benchmark used to parameterize the LC-CUSUM. Changing the benchmark or monitoring horizon could change the crossing point. The case-6 signal should therefore be interpreted as a benchmark-dependent finding, not an intrinsic property of the platform.

Prior reports describe relatively short familiarization periods for experienced surgeons adopting navigated or robotic procedures [20, 22]. In the present study, a current annual volume of approximately 250 knee surgical procedures—including at least 150 robotic procedures—career experience comprising approximately 500 manual UKAs, and extensive prior ROSA^®^ TKA use likely favored a shorter platform-adoption sequence despite the absence of previous robotic UKA experience. This is relevant when extrapolating the case-6 signal to lower-volume or robot-naïve surgeons, but it also allows the present study to isolate familiarization with the new ROSA^®^ Partial Knee workflow from acquisition of basic UKA competence. The secondary cases 1–5 versus 6–30 comparisons were not statistically significant and are interpreted as supportive descriptive analyses rather than independent evidence of temporal improvement.

The standardized single-surgeon workflow reduces procedural heterogeneity but also restricts external validity. The findings may not transfer to surgeons with different UKA volumes, previous robotic exposure, operative-time definitions, patient complexity, or institutional workflows. The case-6 crossing should not be used as a credentialing threshold.

Radiographic measurements were obtained by one observer who was blinded to planned and validated ROSA^®^ values. The 15-case repeat assessment after a 21-day washout quantified intraobserver reliability, but interobserver reliability was not assessed; reproducibility across different readers therefore remains unknown. Projection, positioning, and differences between intraoperative and radiographic measurement systems are reflected in the Bland–Altman limits.

Three-month clinical analyses were restricted to the first 10 chronological cases because the later cases had not reached mature follow-up at extraction. This systematic temporal selection may overestimate or underestimate outcomes if early patients differed from later patients in unmeasured ways; the direction and magnitude cannot be quantified. VAS and KSS were collected in person by the same orthopaedic resident using a consistent assessment procedure. Following verification of the source records, a transcription error in the working database was corrected: at 3 months, six patients had VAS 0 and four had VAS 3, corresponding to a mean of 1.2 ± 1.5. These findings should not be generalized to the complete cohort.

This study has several limitations. Its retrospective, single-surgeon design and 30-case sample limit precision and external generalizability, particularly for Bland–Altman limits and uncommon complications. The LC-CUSUM crossing is conditional on the specified benchmark and monitoring horizon, while the surgeon’s very high procedural volume and previous ROSA^®^ TKA experience likely produced a shorter adoption sequence than might be observed in less-experienced surgeons. The absence of a concurrent manual control group precludes claims of comparative superiority. Clinical follow-up was chronologically limited to the first 10 cases, restricting generalizability of the clinical findings. Finally, femoral component position, sizing, and overhang were not assessed; the radiographic findings therefore concern tibial parameters rather than overall implant accuracy.

A single-surgeon consecutive design reduces variability in surgical technique and permits focused evaluation of adoption of the ROSA^®^ Partial Knee workflow. The surgeon-specific manual-UKA operative-time benchmark was derived from the institutional operating-room register using the same skin-to-skin definition as the present study; the surgeon’s career experience comprising approximately 500 manual UKAs was reported separately to characterize operator experience, not to claim comparative superiority. The chronological 10-case clinical subset resulted from follow-up maturation rather than selective loss, and clinical findings were explicitly secondary. Restriction to tibial radiographic endpoints reflects the parameters directly planned and validated by the studied ROSA^®^ workflow. The study provides an explicitly parameterized LC-CUSUM, formal Bland–Altman analysis, blinded radiographic measurement, exact binomial confidence intervals, and transparent reporting of the prespecified analytical parameters.

In summary, the observed sequence crossed the prespecified temporal-performance boundary at case 6, and postoperative tibial coronal alignment and slope remained within ± 2° of plan in all 30 cases. The result is specific to the surgeon, benchmark, workflow, and endpoints studied.

## Conclusions

The predefined platform-specific temporal-performance standard was reached after six cases by an experienced high-volume UKA surgeon. Tibial planned-to-postoperative deviations were small in this consecutive series, while the findings remain conditional on the specified benchmark and require external validation.

Declaration of generative AI and AI-assisted technologies in the manuscript preparation process: During the preparation of this work, the authors used OpenAI’s ChatGPT as a language-editing tool to improve clarity and readability. After using this tool, the authors reviewed and edited the content as needed and take full responsibility for the content of the published article.

## Data Availability

The datasets generated and analyzed during the current study are not publicly available due to patient privacy and institutional restrictions, but are available from the corresponding author upon reasonable request.

## Abbreviations

BMI: Body mass index
LC-CUSUM: Learning-curve cumulative summation
HKA: Hip–knee–ankle angle
ICC: Intraclass correlation coefficient
KSS: Knee Society Score
ROM: Range of motion
STROBE: Strengthening the Reporting of Observational Studies in Epidemiology
UKA: Unicompartmental knee arthroplasty
VAS: Visual analogue scale
